# Visible-absorption spectroscopy as a biomarker to predict treatment response and prognosis of surgically resected esophageal cancer

**DOI:** 10.1038/srep33414

**Published:** 2016-09-14

**Authors:** Pei-Wen Yang, I-Jen Hsu, Chun-Wei Chang, Yu-Chia Wang, Ching-Yueh Hsieh, Kuan-Hui Shih, Li-Fan Wong, Nai-Yu Shih, Min-Shu Hsieh, Max Ti-Kuang Hou, Jang-Ming Lee

**Affiliations:** 1 Department of Surgery, National Taiwan University Hospital and National Taiwan University College of Medicine, Taipei, Taiwan; 2 Department of Physics and Center for Biomedical Technology, Chung Yuan Christian University, Taoyuan, Taiwan; 3 Graduate Institute of Pathology, National Taiwan University College of Medicine, Taipei, Taiwan; 4 Department of Mechanical Engineering, National United University, Miaoli, Taiwan

## Abstract

The application of optical absorption spectra in prognostic prediction has hardly been investigated. We developed and evaluated a novel two dimensional absorption spectrum measurement system (TDAS) for use in early diagnosis, evaluating response to chemoradiation, and making prognostic prediction. The absorption spectra of 120 sets of normal and tumor tissues from esophageal cancer patients were analyzed with TDAS *ex-vivo*. We demonstrated the cancerous tissue, the tissue from patients with a poor concurrent chemoradiotherapy (CCRT) response, and the tissue from patients with an early disease progression each had a readily identifiable common spectral signature. Principal component analysis (PCA) classified tissue spectra into distinct groups, demonstrating the feasibility of using absorption spectra in differentiating normal and tumor tissues, and in predicting CCRT response, poor survival and tumor recurrence (efficiencies of 75%, 100% and 85.7% respectively). Multivariate analysis revealed that patients identified as having poor-response, poor-survival and recurrence spectral signatures were correlated with increased risk of poor response to CCRT (P = 0.012), increased risk of death (P = 0.111) and increased risk of recurrence (P = 0.030) respectively. Our findings suggest that optical absorption microscopy has great potential to be a useful tool for pre-operative diagnosis and prognostic prediction of esophageal cancer.

Esophageal cancer is a deadly disease with high risk of local recurrence and distant metastasis even among patients with early stage tumors. Primary esophageal cancer presents most often as esophageal squamous cell carcinoma (ESCC) or adenocarcinoma (EADC)^1^,^2^,^3^. The standard treatment for locally advanced esophageal cancer is neoadjuvant (preoperative) concurrent chemoradiotherapy (CCRT) with or without surgery. Patients with ESCC can enjoy better survival once they have shown a good response to CCRT^4^. The pathologically complete remission rate has only ranged from 10% to 40% under different treated protocols^5^,^6^,^7^. Prognosis of esophageal cancer is still poor with a 5-year survival rate of less than 20% even with multiple treatment modalities^1^,^2^,^6^,^8^. More than 50% of the patients encounter local-regional recurrence or distant metastases within 2 to 3 years^9^,^10^,^11^. The TNM staging has been considered the gold standard in predicting clinical outcome and guiding treatment strategy^12^,^13^,^14^. However, some patients even with early stage disease experience local or systemic failure early after treatment^15^,^16^,^17^,^18^,^19^. Therefore, an effort is underway to identify and make use of multiple reliable prognostic markers to improve management of patients with esophageal cancer.

Optical spectroscopy is a technique that explores the properties of physical objects based on their interaction with light, where the spectroscopic data is usually represented by a spectrum. Because the optical spectrum of a tissue contains information about the structure and the biochemical composition of the tissue that can be accessed non-invasively and in real-time, optical spectroscopy has become an important technique in the diagnosis of cancers^20^. The types of spectroscopy can be classified by the nature of the interaction between the object and the energy, including resonance, absorption, emission, elastic scattering (reflection), and inelastic scattering^21^. The resonance method has been productively used in nuclear magnetic resonance (NMR) spectroscopy.

Absorption spectroscopy is based on the absorption of the radioactive source by the material and is often analyzed by measuring the transmitted fraction of the energy through the material. Absorption spectroscopy is useful for distinguishing the molecular components of tissue according to the different absorption properties of the biomolecules comprising them, which is accomplished by measuring absorption spectra^22^. There are a number of major molecules known to contribute to the absorption spectrum of biological tissues in specific ways. In the UV region, protein and DNA result in increased absorption of shorter wavelengths. The molecules of endogenous chromophores, including tryptophan, NAD+ (Nicotinamide adenine dinucleotide), collagen, elastin, NADH, and FAD (Flavin adenine dinucleotide) are also important absorbers in biological tissue^23^. Increased absorption of longer wavelengths in the near infrared region (NIR) is mostly due to tissue water content^22^. In the visible and NIR regions, the ground states of oxy- and deoxyhemoglobin contribute to absorbance, allowing for evaluation of tissue oxygenation. The absorption spectrum signatures of deoxygenated hemoglobin (Hb) and oxygenated hemoglobin (HbO_2_) are quite distinct^24^,^25^. Other chromophores such as melanin (from melanosomes) and cytochrome may also contribute to the endogenous absorbance of tissue. Myoglobin derivatives are also strong absorbers in tissue. The absorption maxima of oxymyoglobin, reduced myoglobin, metmyoglobin and nitrosomyoglobin are localized within the 410 to 640 nm range of the visible region^26^. In the region of 600–1000 nm, scattering predominates over absorption in tissue and has been described as the diagnostic and therapeutic window^27^.

Traditional “biopsy” refers to the removal of tissue for diagnostic evaluation, whereas “optical biopsy” usually indicates that evaluation is done on tissue that is not removed^20^. The evaluation of tissue pathology by “optical biopsy” has some advantages due to its non-invasiveness and real-time *in-situ* assessment. Pathology reports from biopsy are currently an essential guideline to predict prognosis. However, current histopathology assessment usually requires invasive surgical removal of the tissue as *in vivo* techniques are difficult to perform. Magnetic resonance spectroscopy (MRS) data of fine-needle aspiration taken from breast tumor has been demonstrated to make it possible to distinguish malignant tissue with over 93% accuracy^28^. Notably, Raman microscopy can differentiate malignant lung tissue from non-malignant bronchial epithelium, and has the power to predict early recurrence in patient receiving lung surgical resection^29^. Recently, Raman spectroscopy of post serum samples has also been suggested to have the potential to predict recurrence in oral cancers^30^. Research, thus, has clearly shown the great potential for using optical spectroscopy data in predicting and evaluating clinical outcome.

The application of optical absorption spectra in cancer diagnosis and prognostic prediction has never been investigated. In the current study, an optical system for the detection of the absorption spectra of human esophageal tissue in the visible range (450–650 nm) was designed and established. The absorption spectra of both normal and cancerous esophageal tissue biopsy specimens from 120 patients with primary esophageal cancer were analyzed and correlated with CCRT response and clinical outcome.

## Results

### Characteristics of patients

The characteristics of the 120 enrolled patients who received surgical resection for primary esophageal cancer are listed by fresh (n = 56) or frozen (n = 64) analysis in Table 1. Of these patients, 93.3% (n = 112) were male, 94.2% (n = 113) were with ESCC, and 68.3% (n = 82) were without lymph node metastasis. The location of tumors in the upper, middle and lower third of the esophagus were 28 (23.3%), 44 (36.7%) and 48 (40.0%) respectively. There were 103 (85.8%) patients treated with CCRT [CCRT (+)] of which 40 (38.8%) had complete remission.

### Median absorption spectra of CCRT (−) esophageal tissues

**Figure 1:**
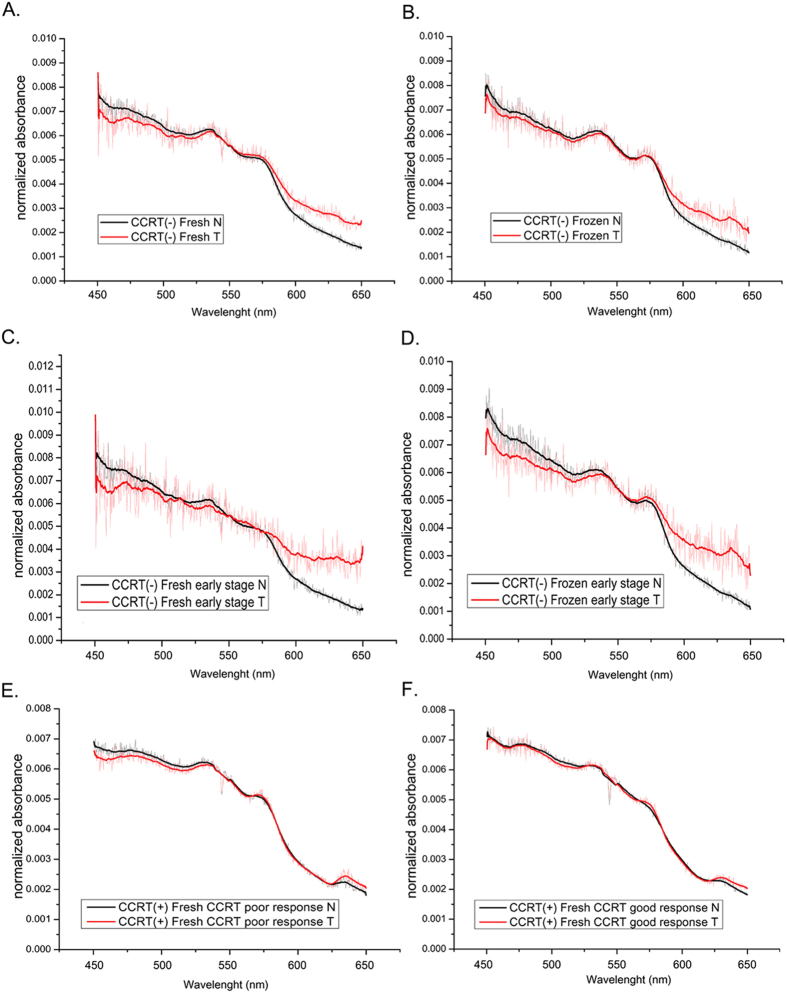
Median absorption spectra of fresh tissue (**A,C,E,F**), frozen tissue (**B,D**) tissue without CCRT treatment (**A–D**) or tissue with CCRT treatment (**E,F**). (**C,D**) Tissue from early-stage patients. (**E,F**) Tumor tissue from poor responders (**E**) or good responders (**F**) to CCRT. N, normal; T, tumor.

Median absorption spectra of normal and malignant tissue specimens from patients treated without CCRT [CCRT(−)] and analyzed by TDAS freshly are presented in Fig. 1A. The spectra of the malignant tissue showed lower absorption in short-wavelengths (around 450–500 nm) and higher absorption in long-wavelengths (around 575–650 nm) compared with the median spectra of normal tissue. Similar results were observed in the frozen tissue (Fig. 1B). These features were also evident in the tissue specimens from patients with early tumor stage (Fig. 1C,D). Interestingly, the freshly analyzed tumor tissue from poor responders to CCRT also displayed lower absorption in short wavelengths (around 450–500 nm) and higher absorption in long wavelengths (around 625–650 nm) in comparison with the spectrum of CCRT-treated normal tissue (Fig. 1E). These features were less obvious in tissue from good responders (Fig. 1F).

### Median absorption spectra of CCRT (+) esophageal tissues

**Figure 2:**
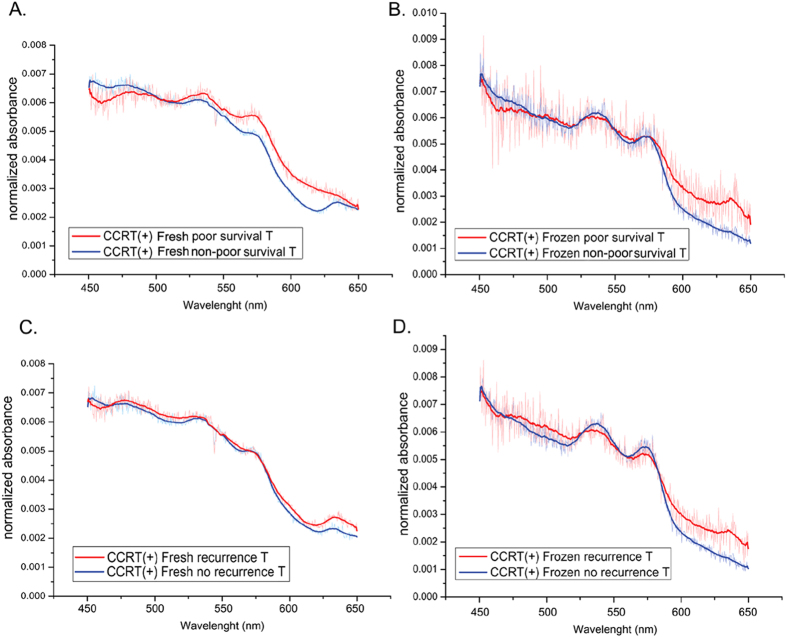
Median absorption spectra of fresh (**A**,**C**) or frozen (**B,D**) tissue samples treated with CCRT. (**A,B**) Tissues from non-poor or poor survival groups. (**C,D**) Tissues from recurrence or no recurrence groups. T, tumor.

The median spectral signatures of tumor tissue also displayed notable differences between non-poor survival and poor survival groups. In the fresh CCRT (+) tumor tissue samples, the median spectra of patients with poor survival also showed reduced absorption in short wavelengths (around 450–500 nm) and enhanced absorption in long wavelengths (around 500–650 nm) compared with the spectra of the non-poor survival group (Fig. 2A). Similar results were obtained in the frozen tissues (Fig. 2B). Spectral analysis of fresh tumor also showed higher absorption in long wavelengths (around 600–650 nm) among the recurrence group compared with the no-recurrence group (Fig. 2C). Parallel analysis of frozen tissue revealed a similar result (Fig. 2D).

### Median absorption spectra of ESCC and EADC tissues

We then analyzed the spectral signatures of ESCC (n = 113) and EADC (n = 5) tissue. The median spectra of normal and tumor tissue samples from EADC patients showed significantly lower absorption in long-wavelengths (around 625–650 nm) compared with the median spectra of tissue samples from ESCC patients (Fig. S2).

### Classification of esophageal tissue spectra by PCA

We further analyzed the spectral signatures by principal component analysis (PCA), a commonly used statistical method of variable reduction in spectral analysis^31^. Because we hope to establish a prediction model for fresh sample analysis, we enrolled all the fresh samples (n = 56) for the PCA analysis. We also included CCRT (−) frozen samples (n = 10) in the PCA analysis due to the limited fresh samples of CCRT (−) tissues (n = 7). There were a total of 66 cases included for PCA. The total spectral data consist of 132 sets, 66 of normal tissue spectra and 66 of tumor tissue spectra. There were 900 intensity values in the raw data of each spectrum in the wavelength range of 450–650 nm which were defined as the spectral variables. The wavelengths of 450 to 475 nm and of 625 to 650 nm were found by independent t-test to clearly divide the patients into different groups, including normal and tumor groups, good response and poor response groups, non-poor survival and poor survival groups, and recurrence versus no recurrence groups (data not shown). PCA thus extracted 150 spectral variables from the wavelengths of 450 to 475 nm and of 625 to 650 nm. The first eight PCs (principal components) were able to describe about 79% of the variation in the spectral data from all fresh samples and CCRT (−) frozen samples.

### Identification of tumorous spectra and non-tumorous spectra by PCA

**Figure 3:**
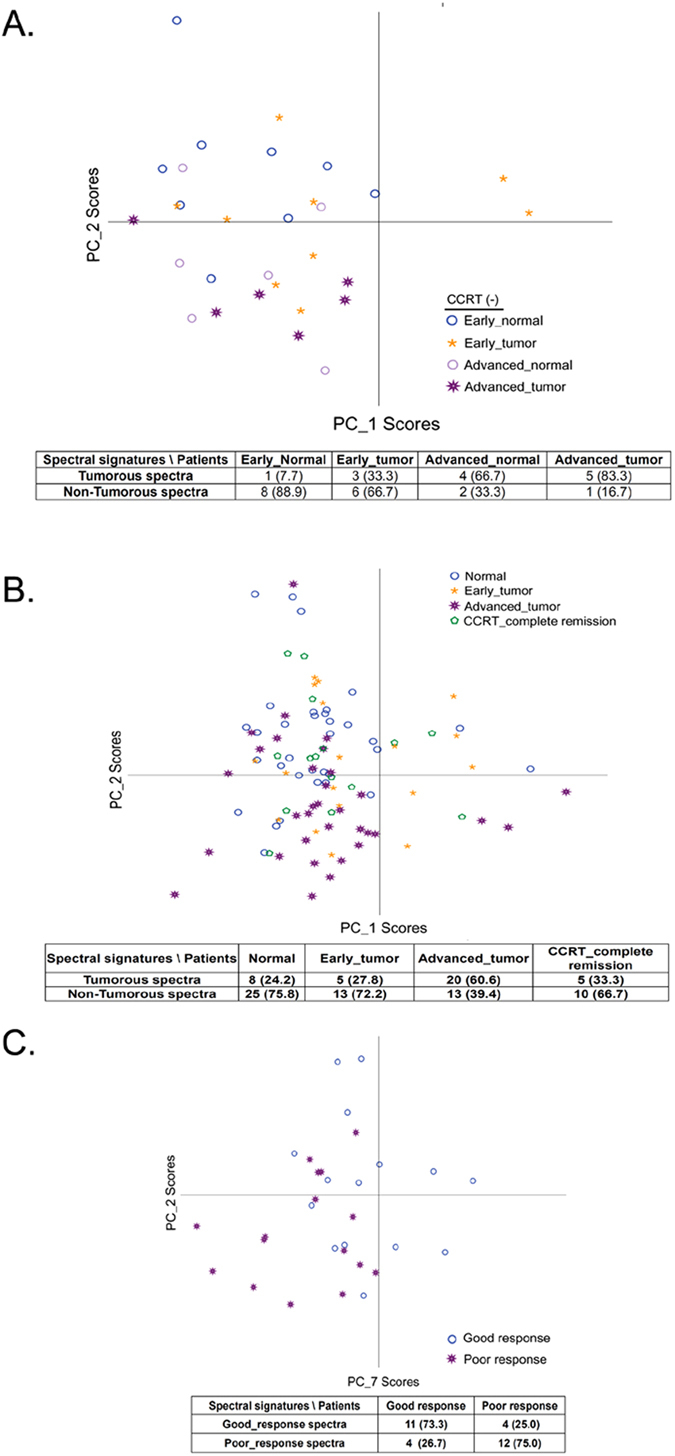
(**A,B**) Spectral signatures, tumorous spectra or non-tumorous spectra, classified by the scatter plots of PC_1 and PC_2 scores from PCA. (**A**) CCRT (−) tissues (**B**) CCRT (−) and CCRT (+) -complete remission tissues. (**C**) Spectral signatures of CCRT (+) tissues, good-response spectra and poor-response spectra, classified by the scatter plots of PC_7 and PC_2 scores from PCA. PC, principal component; PCA, principal component analysis.

We initially included the spectra of CCRT (−) ESCC cases (n = 13 for both normal and tumor tissue) to establish the normal/tumor prediction model by PCA. The first 2 components, PC_1 and PC_2, were able to efficiently identify early-normal (normal tissue from early-stage patients) spectra and early-tumor (tumor tissue from early-stage patients) spectra and could distinguish with 83.3% sensitivity the advanced-tumor spectra (Fig. 3A). We then included the rest of spectral data sets, including the CCRT (+) tissue spectra (total number of spectral data sets n = 132), but excluded adjacent normal tissue samples of patients with advance tumor stages (advanced-normal spectral data sets n = 33) because of the likelihood that the advanced normal tissue might be invaded by tumor. The absorption spectra make it possible to differentiate advanced tumor and CCRT complete remission tissue from normal tissues with a sensitivity of 60.6% and a specificity of 66.7% respectively (Fig. 3B). However, the sensitivity was only 27.8% in distinguishing early stage tumor tissue from normal tissue (Fig. 3B). We classified the median spectra into tumorous spectra and non-tumorous spectra by the groupings of the PC_1 and PC_2 loading plots. The tissue samples with tumorous spectra displayed a 2.59-fold increased odds of being malignant compared with the tissue samples with non-tumorous spectra (Logistics regression model, OR [95% CI] = 2.59 [1.18–6.00], P = 0.027, Table 2).

### Spectral signatures of good response and poor response to CCRT

We then examined whether the absorption spectra could be used to evaluate CCRT response in esophageal cancer by including the 31 cases treated with CCRT for which a clear pathologic response record existed. It was found that the values of PC_7 and PC_2 could be used to differentiate poor responders and good responders with a sensitivity of 75% and a specificity of 73.3% (Fig. 3C). Patients identified as having a poor-response spectra showed a significant, more than 11-fold increased risk of poor response compared with patients identified as having a good-response spectra (Logistics regression model, OR [95% CI] = 11.69 (1.73–79.20), P = 0.012, Table 2).

### Optical spectra predict overall survival and early disease recurrence of esophageal cancer

**Figure 4:**
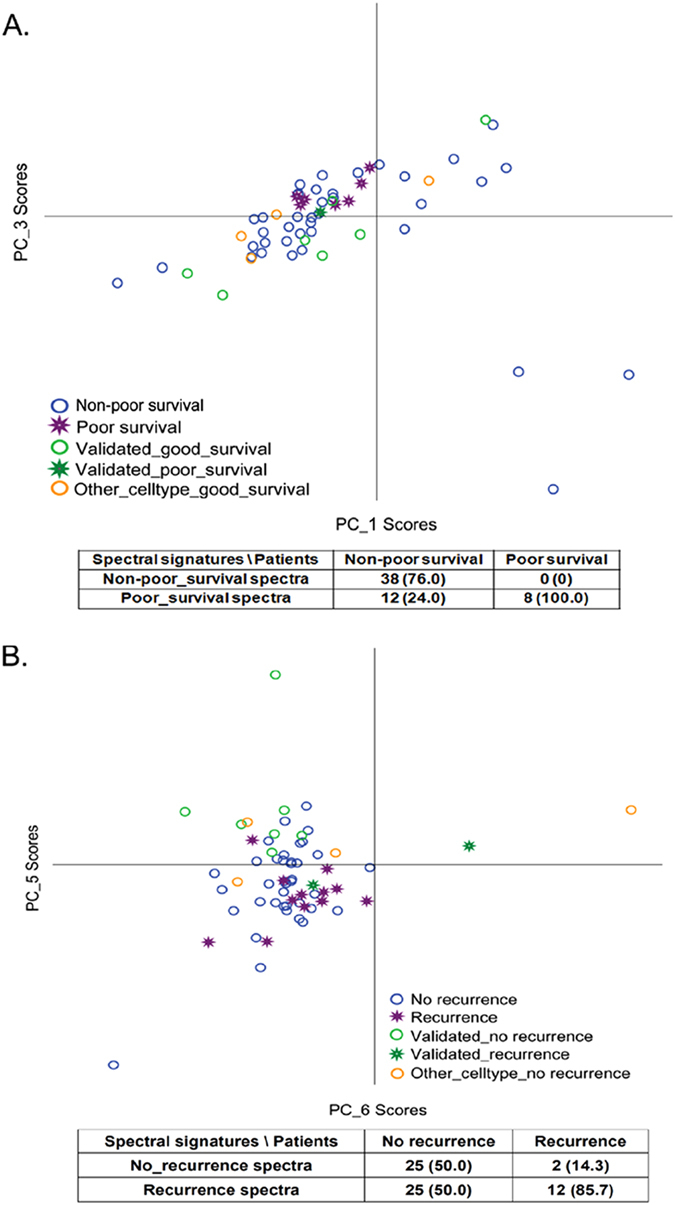
PCA of tumor tissue samples. (**A**) The spectral signatures of non-poor-survival spectra and poor-survival spectra by the scatter plots of PC_1 and PC_3 scores and (**B**) The spectral signatures of no-recurrence and recurrence spectra by the scatter plots of PC_6 and PC_5 scores.

Raman microscopy can be used to predict disease recurrence in solid tumors, including lung and oral cancers after surgical resection^29^,^32^. We thus evaluated whether absorption spectroscopy data might serve as a biomarker to predict the prognosis of surgically resected esophageal cancer using PCA to analyze the cases with a follow up of more than 12 months (n = 58 for survival analysis) or more than 6 months (n = 64, for recurrence analysis). The median spectra from fresh ESCC tissue were assigned to a training data set. The scatter plot constructed by PC_1 and PC_3 scores perfectly separated the poor survival group from the non-poor survival group with a sensitivity of 100% (Fig. 4A). Frozen CCRT (−) ESCC samples (n = 7, 6 non-poor survival and 1 poor survival) and tissue of other histological types (n = 4, all non-poor survival) were assigned to a testing set for validation. The tissue from the patient with poor survival in the testing set was clustered with the poor survival group in the training set (Fig. 4A). 7 of the 9 patients in the testing set with non-poor survival displayed non-poor survival spectra and were classified properly (Fig. 4A). The specificity for survival prediction was 76% combing training and testing group. We then analyzed the association between spectral signature and early recurrence of patients. Patients who experienced tumor recurrence or died within 6 months after surgery were assigned to the recurrence group in the current study. The PC_6 and PC_5 scores efficiently separated the recurrence group from the no recurrence group in the training set (Fig. 4B). In the testing set, 2 patients had early recurrence and 11 were recurrence-free. One patient with early recurrence (50%) and 10 patients (91%) without early recurrence were classified correctly (Fig. 4B). Overall, the sensitivity and specificity for predicting recurrence were 85.7% and 50% respectively.

**Figure 5:**
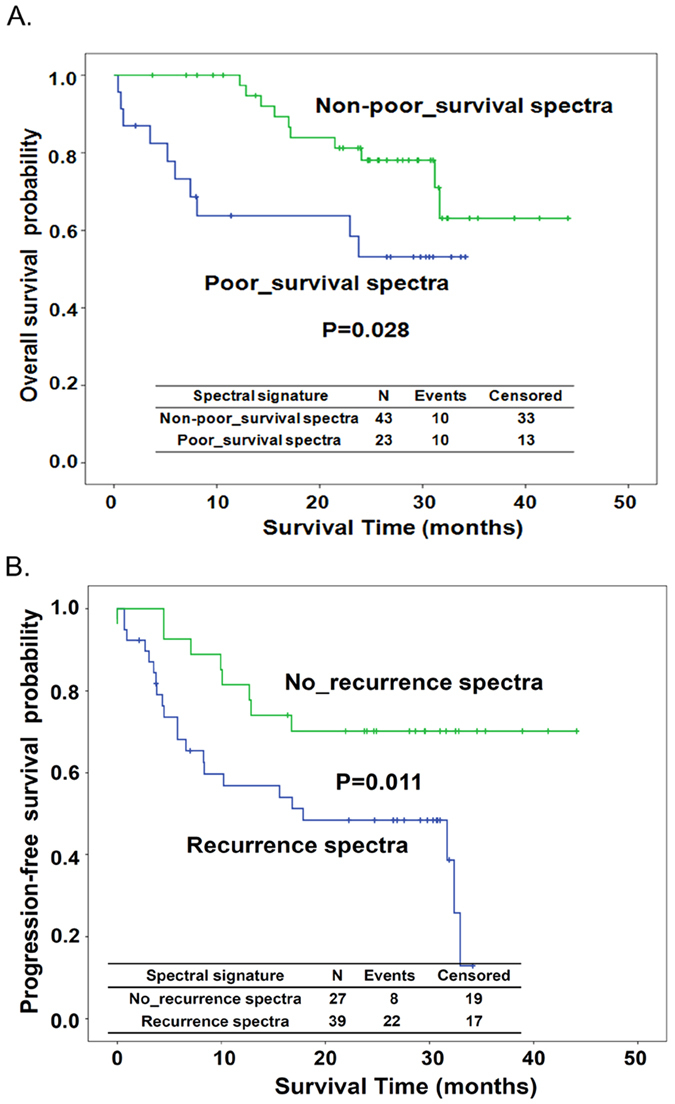
Kaplan-Meier estimates of overall survival by the spectral signatures of non-poor-survival spectra and poor-survival spectra (**A**), and progression-free survival by the spectral signatures of no-recurrence spectra and recurrence spectra (**B**).

Under multivariate analysis, patients with poor survival spectra showed a trend of correlation with increased risk of death compared to patients with non-poor survival spectra (Cox regression model, HR [95% CI] = 2.48 (0.81–7.57), P = 0.111, Table 3). Meanwhile, patients with recurrence spectra had a statistically significant, 2.78 fold higher relative risk of disease recurrence (Cox regression model, HR [95% CI] =  2.78 (1.10–7.00), P = 0.030, Table 3). The Kaplan-Meier method also demonstrated that differences in overall survival (OS) and progression-free survival (PFS) showed corresponding differences in spectral signature. Patients with poor survival spectra exhibited significantly shorter overall survival compared to those with non-poor survival spectra (log-rank P = 0.028, Fig. 5A). As expected, those with recurrence spectra were significantly associated with reduced progression-free survival rate compared to those with no-recurrence spectra (log-rank P = 0.011, Fig. 5B).

## Discussion

Cancer formation is usually triggered by accumulating genetic or epigenetic mutations, which result in great changes in the biochemical compounds in cells. Different degrees of malignancy are accompanied by different molecular compounds in individual cancer cells, which in turn call for different therapeutic strategies. Current technologies, such as microarrays, are widely used to analyze global differences in biological materials between individual samples^33^. These analytic platforms provide detailed information about global changes in gene expression; however, they are not designed for real-time *in-situ* assessment and require considerable time and cost.

Absorption spectroscopy is a potentially powerful tool for cancer diagnosis due to its ability to provide accurate integral absorption information about the sample in real-time or *in-situ*. It has been used to detect the absorption spectra covering the range of wavelengths from ultraviolet (UV) through visible to near-infrared (NIR) in the tissue of human breast cancer xenografts implanted in nude mice^34^. The breast cancer tissue was observed to display an Hb signature while normal tissue exhibited a signature similar to HbO_2_. Since the concentration of blood oxygen around tumors is frequently lower than the concentration in normal tissue due to the excessive need of cancer cells for oxygen^25^,^35^, the absorption spectra from the breast tissue provides valuable and reasonable information for the cancer diagnosis.

Visible light is safe and easy to work with for clinical diagnosis. Our experience suggested that the visible absorption spectral signatures in esophageal normal and tumor tissues should be distinct enough to provide valuable diagnostic information. Our current pilot study systematically integrated the fields of biomedical optics, surgery, pathology and mechanical engineering to analyze the visible absorption spectral signatures of esophageal tissue from patients with esophageal cancer using an optical system that we specifically designed for tissue sample analysis. We demonstrated the discrimination of the spectral signatures in normal and tumor tissues (Fig. 1); and the tissues from patients with different clinical outcomes, including CCRT response, survival and recurrence (Figs 1 and 2). We also observed a significant difference between the spectral signatures of ESCC and EADC in long- wavelengths even though the number of EADC samples was limited (Fig. S2).

Concerns might be raised that absorption microscopy in the transmission mode cannot be applied reliably for *in-situ* cancer diagnosis because it is not possible to effectively detect light penetrated through the human body. In fact, the TDAS was designed to be able to measure an absorption spectrum in both the transmission and reflection modes (Fig. S1). We established a prediction model by using the data we collected in the transmission mode because of their higher signal-to-noise ratio compared to the data we collected in the reflection mode. Our results revealed that the absorption spectra measured in the reflection mode were almost identical to those measured in the transmission mode, though they exhibited a slightly lower sensitivity (Fig. S3), which indicates the possibility that absorption microscopy performed in reflection mode could also provide real-time/*in-situ* diagnosis by integrating it with an endoscope.

As in the breast cancer study^34^. the relative absorption spectra of esophageal tumor tissue showed a signature similar to the spectra of Hb, with weaker absorption in the interval of 450 to 550 nm and stronger absorption in the light region above 600 nm compared to the signature of HbO_2_^24^. Interestingly, the tumor tissue spectra from patients with adverse clinical outcomes were also more similar to an Hb spectral signature than the tumor spectra from those with favorable clinical outcomes. Hypoxia is a key factor for tumor formation^36^. We preliminarily found that hypoxia-inducible factor-1α (HIF-1 α) was significantly expressed in the esophageal tumor tissue of 6 of the 10 cases with esophageal cancer (Fig. S4). The surface oxygen level of paired normal and tumor tissues were also analyzed. Tumor oxygen levels were lower than the oxygen levels in normal tissues, though without reaching statistical significance (P = 0.289, N = 25, Fig. S5). We thus propose that the difference in the absorption properties between normal and malignant esophageal tissues results partially from the difference in the oxygen content of the tissues. It has been found that increased hemoglobin concentration correlates with decreased risk of death in patients after radical treatment of esophageal cancer with radiotherapy^37^. Since total hemoglobin level usually includes the oxyhemoglobin form (HbO_2_) and the deoxyhemoglobin form (Hb), whether a high level of HbO_2_ but not of Hb is associated with adverse prognosis in esophageal cancer patients needs further investigation.

We also preliminarily explored possible cellular factors that might influence the absorption property of tissue by analyzing global gene expression changes of tumor tissue relative to normal tissue using DNA microarray analysis. As expected, the gene expression change for each of the absorption factors, including cytochrome P450, elastin, melanin, and myoglobin related genes, was more significant and extensive in tumor tissue with a distinct spectral signature than in tumor tissue without the distinct spectral features (data not shown).

Accurate pre-operative and post-operative diagnosis of CCRT response usually requires pathologic examination of tissue samples obtained from endoscopic ultrasound (EUS)-guided biopsy and surgical resection. Since accurate pathologic diagnosis relies on adequate and correct sampling of the lesion sites, we believe spectral information obtained during endoscopic examination will help physicians evaluate esophageal lesions more easily and accurately. The optical spectra of CCRT treated tissues have never been studied; we analyzed the spectral signature in tissues from good responders and poor responders. PCA successfully differentiated poor responders from good responders with a sensitivity and specificity of 75% (Fig. 3C). There was an 11-fold increased risk of poor response for the patients with poor-response spectra compared to patients with good-response spectra (Table 2). It has been found that the gene expression signature differs between patients with pathological complete remission (pCR) and those less than pCR in response to CCRT^38^. Our results suggest that the content of absorptive components, such as cytochrome C, a key factor in apoptosis^39^, might be sufficiently distinct between good-response and poor-response tissue to allow reliable discrimination of their spectral signatures.

Esophageal cancer is a deadly disease with high risk of recurrence^9^,^10^,^11^. The median survival after recurrence of ESCC is reportedly only about 8 months^11^.

Multiple reliable prognostic markers are benefit for the improvement of therapeutic outcome of esophageal cancer. We previously reported that the single nucleotide polymorphisms (SNPs) at the genes involved in the nucleotide excision repair and growth factor-related pathway can be prognostic biomarkers of esophageal cancer^40^,^41^,^42^,^43^. Our current study demonstrates the feasibility of using visible absorption spectra to predict survival and recurrence of patients with esophageal cancer, backed by results from PCA, multivariate regression model, and Kaplan-Meier estimate (Figs 4 and 5 and Table 3). The potential of Raman spectroscopy to predict recurrence in cancers, based on its ability to detect the molecular heterogeneity of tumors, has been suggested and demonstrated^30^,^44^. We found that the spectral features of tissue from patients with adverse clinical outcome tend to be similar to the spectral features of deoxygenated hemoglobin.

Hypoxia is also an adverse prognostic factor in various cancers, including in loco-regional gastroesophageal cancer^45^. It induces extensive alteration in expression of prognostically relevant factors, such as hypoxia-inducible factor-1 (HIF-1)^46^ and IGFBP3^21^. In our microarray analysis, we observed that expression of a gene, MMP7 (matrix metallopeptidase 7,Matrilysin), exhibited more than a 60-fold increase in the tumor tissue with a distinct spectral signature while only a 3-fold increase in the tumor tissue without the distinct spectral signature, both in comparison to expression levels in normal tissue (data not shown). MMP7 is a member of the MMPs (matrix metalloproteinases). It is frequently over-expressed and associated with tumor progression and metastasis in human cancer, including esophageal cancer^47^,^48^,^49^,^50^. MMP7 has been found to be induced by hypoxia in macrophages^51^. Whether hypoxia is correlated with tumorous and adverse prognostic spectral signatures and whether it is correlated with over-expression of MMP7 in esophageal tissues needs further investigation.

In the current study, we investigated use of the TDAS system only for the analysis of esophageal tissue from patients diagnosed with primary esophageal cancer. However, TDAS might also be useful in the diagnosis and treatment of more benign diseases of the esophagus, such as benign tumors, inflammation, lesions and polyps. It would be of interest to systematically analyze the spectral signatures of esophageal tissue in patients with these benign diseases using larger sample sizes.

TDAS shows less sensitivity in differentiating normal-to-early stage transition than in prognostic prediction. We speculate that one of the reasons for this is that we established the tumor prediction model using CCRT (−) ESCC samples, of which there were only 13 cases. Another point of interest is that our current analytic method of using median spectral analysis gave the entire results of the scanning range (5 mm × 5 mm) for each sample, which made the signal strength strongly correlated with the size of tumor. The spectral signal power might have been diminished in early-tumor tissue due to the relatively small mass of the early-stage tumor tissue. We will now work on creating an algorithm for the TDAS system which can efficiently evaluate the probability of malignancy in each single mass of tissue, and thereby improve the accuracy of its prediction model.

In conclusion, our study is the first to demonstrate the potential of visible-absorption spectroscopy as a tool for evaluating CCRT response and as a prognostic biomarker of esophageal cancer. A larger sample size of spectral information would in all likelihood make the prediction model more accurate. We also need an animal model to prove the feasibility of TDAS for *in-vivo* diagnosis in the future.

## Methods

### Study population and tissue collection

A total of 120 patients treated with surgical resection for primary esophageal cancer at National Taiwan University Hospital (NTUH) from 2011 to 2013 were enrolled in the study. Tissue sets, including tumorous and non-tumorous (normal, distant from the tumor) samples of esophageal tissue, were collected during surgical dissection. Of these specimens, 56 sets were fresh tissues analyzed to determine their optical spectrum within 1 hour after tissue dissection. The remaining 64 sets were frozen tissues which were stored in a −80 °C freezer and analyzed within 1 year. Patients were followed up in our outpatient clinics. Information about patients, including demographics, histology, TNM stage, treatment response, survival and recurrence, was obtained by medical chart review. This study was approved the Research Ethics Committee of NTUH (No. 201101065RB). Written informed consent was obtained from all participating subjects. All the study methods were carried out in accordance with the approved guidelines.

### Two dimensional absorption spectrum measurement system (TDAS) and spectral analysis

An optical system for measurement of the transmission absorption spectrum was designed and built as shown in Fig. S1. The system is assembled with three components: one for illumination, one for positioning and one for detection. The illumination component comprises an achromatic focusing lens and a collimating lens coupled with an optical fiber attached to a tungsten halogen light source (Ocean Optics, HL2000-HP-FHSA) with a wavelength range from 360 nm to 1700 nm and an output power of 20 W. The positioning component is a sample stage attached to a two-dimensional translation stage (SIGMA KOKI, SGSP15-10XY) for positioning and scanning the sample. The detection part comprises a collecting lens, a reflection prism and a collimating lens coupled to a spectrometer (Ocean Optics, USB2000) via an optical fiber after passing through a neutral-density filter. The main structure of the system is made of polymethyl methacrylate and the whole system is automatically controlled and synchronized with a computer program. The sample stage is moved by the computer-controlled two-dimensional translation stage to perform a two dimensional scan of the sample to be examined. The light transmitted through the sample is collected by the collecting lens and reflected by the reflection prism at a right angle before being coupled to the optical fiber with the collimating lens. The transmission spectrum is detected by the fiber-coupled spectrometer which is automatically triggered by a computer where the signal is processed. The scanning range of each sample is 5 mm × 5 mm with a step size of 500 μm. The absorbance at each point of the sample is given by


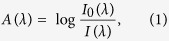


where *I*_0_(λ) is the spectrum of the incident light and *I* (λ) is the measured transmission spectrum. Because the measured spectra show more significant signals and differences in wavelength range between 450 nm and 650 nm, each absorbance is windowed in this wavelength range and is intensity-shifted so that the minimum value is zero, in order to obtain the relative absorbance. The relative absorbance is then normalized so that the area under the spectrum curve is unity to obtain the normalized relative absorbance.

### Data analysis

The noise spectra, including glass-like spectra, heterogenesis spectra, and outlier signals, were removed using the *R* statistical package (https://www.r-project.org/), and the resulting absorption spectra were quantile normalized using the limma package. Median values of both the non-tumor spectra (spectra of normal tissue) and tumor spectra (spectra of tumor tissue) were analyzed, and the median spectra were constructed using Origin software (OriginLab, Northampton, MA, USA) and compared by independent t-test. To further analyze the spectral signatures between groups, the spectral data were grouped by applying principal component analysis (PCA). The associations between spectral signatures and tumor development or CCRT response were described by odds ratios (ORs) obtained from logistic regression. Hazard ratios (HRs) of death and disease recurrence obtained by multivariate Cox regression analysis were used in analyzing the correlation between spectral features and prognosis, including survival and disease recurrence in patients. Crude correlations between spectral groups and survival or disease recurrence were estimated by the Kaplan-Meier method and log-rank test.

In the current study, patients with a complete pathological response or microscopic residual disease after CCRT were classified as “good responders” to CCRT, whereas those with macroscopic residual disease or progressive disease after treatment were classified as “poor responders” to CCRT. Those who were alive over 1-year (12 months) after surgery were considered as having “non-poor survival” whereas patients who died within 1 year after surgery were defined as having “poor survival”. “Recurrence” was defined as patients who died or had detectable tumor recurrence within 6 months. Patients who were recurrence-free over 6 months after surgery were placed in the “no recurrence” group. Overall survival (OS) was defined as the time interval from surgical removal of esophageal tumor (esophagectomy) to the last follow-up or death from disease. Progression-free survival (PFS) was defined as the time elapsed between esophagectomy and death or detection of disease recurrence, including local recurrence or distant metastasis, of the tumor. The statistical analyses were performed by SPSS 16.0 (SPSS Inc, Chicago, IL, USA). A *p*-value less than 0.05 was reported as statistically significant.

## Additional Information

**How to cite this article**: Yang, P.-W. *et al*. Visible-absorption spectroscopy as a biomarker to predict treatment response and prognosis of surgically resected esophageal cancer. *Sci. Rep.* **6**, 33414; doi: 10.1038/srep33414 (2016).

## Supplementary Material

Supplementary Information

## Figures and Tables

**Table 1:** Characteristics of patients.

	N	Fresh	Frozen
		56 (46.7)	64 (53.3)
Histology			
ESCC	113 (94.2)	52 (92.9)	61 (95.3)
EADC	5 (4.2)	4 (7.1)	1 (1.6)
others	2 (1.7)	0 (0)	2 (3.1)
Gender			
Female	8 (6.7)	3 (5.4)	5 (7.8)
Male	112 (93.3)	53 (94.6)	59 (92.2)
Age		57.95±8.74	56.14±10.01
T-stage			
0	35 (29.2)	15 (26.8)	20 (31.3)
1	33 (27.5)	11 (19.6)	22 (34.4)
2	24 (20.0)	13 (23.2)	11 (17.2)
3	28 (23.3)	17 (30.4)	11 (17.2)
N-stage			
0	82 (68.3)	36 (64.3)	46 (71.9)
1	29 (24.2)	17 (30.4)	12 (18.8)
2	4 (6.3)	3 (5.4)	4 (6.3)
3	2 (3.1)	0 (0)	2 (3.1)
Site			
upper	28 (23.3)	13 (23.2)	15 (23.4)
middle	44 (36.7)	16 (28.6)	28 (43.8)
lower	48 (40.0)	27 (48.2)	21 (32.8)
CCRT			
0	17 (14.2)	7 (12.5)	10 (15.6)
1	103 (85.8)	49 (87.5)	54 (84.4)
CCRT response			
Complete remission	40 (38.8)	15 (30.6)	25 (46.3)
Microscopic tumor	30 (29.1)	18 (36.7)	12 (22.2)
Macroscopic tumor	33 (32.0)	16 (32.7)	17 (31.5)

ESCC, esophageal squamous cell carcinoma.

EADC, esophageal adenocarcinoma.

CCRT, concurrent chemoradiotherapy.

**Table 2:** Association of spectra groups with tumor risk and CCRT response under multivariate analysis.

Variables	N	OR (95% CI)	*P-value
Tumorous			**0.027**
Non-tumorous spectra	61	1	
Tumorous spectra	38	2.59 (1.18–6.00)	
CCRT_response			0.012*
Good_response spectra	15	1	
Poor_response spectra	16	11.69 (1.73–79.20)	

*Adjusted for cell type and site.

**Table 3:** Association of spectra groups with overall or progression-free survival of esophageal cancer patients under multivariate analysis.

Variables	N	HR (95% CI)	*P-value
Overall Survival			0.111*
Non-poor_survival spectra	43	1	
Poor_survival spectra	23	2.48 (0.81–7.57)	
Progression-free Survival			
No_recurrence spectra	27	1	0.030*
Recurrence spectra	39	2.78 (1.10–7.00)	

*Adjusted for T_stage, N_stage, site, CCRT treatment and cell type.
