# Supplementary material for: Visible-absorption spectroscopy as a biomarker to predict treatment response and prognosis of surgically resected esophageal cancer
Source: Sci Rep. 2016 Sep 14;6:33414. doi: 10.1038/srep33414 (PMC5022060; doi:10.1038/srep33414)
Supplement: Supplementary Information [file srep33414-s1.pdf]

Visible-absorption spectroscopy as a biomarker to predict treatment response and prognosis of surgically resected esophageal cancer

Pei-Wen Yang[1*] , **I-Jen Hsu**[2*#], **Chun-Wei Chang,**[2] **Yu-Chia Wang,**[2] **Ching-Yueh Hsieh**[1], **Kuan-Hui Shih**[1], **Li-Fan Wong**[1], **Nai-Yu Shih**[1], **Min-Shu Hsieh**[3] , **Max Ti-Kuang Hou**[4], and **Jang-Ming Lee**[1#]

[1]Department of Surgery, National Taiwan University Hospital and National Taiwan University College of Medicine, Taipei, Taiwan
[2]Department of Physics and Center for Biomedical Technology, Chung Yuan Christian University, Taoyuan, Taiwan
[3]Graduate Institute of Pathology, National Taiwan University College of Medicine, Taipei, Taiwan
[4]Department of Mechanical Engineering, National United University, Miaoli, Taiwan

*These authors contributed equally to this work

#Correspondence should be addressed to:

Prof. Jang-Ming Lee, email: jmlee@ntu.edu.tw;

Prof. I-Jen Hsu, email: ijhsu@cycu.edu.tw

Running title: Absorption spectrum in esophageal cancer

Conflicts of interest: There is no conflict of interest for any author regarding the publication of this manuscript.

(A) **(B)**

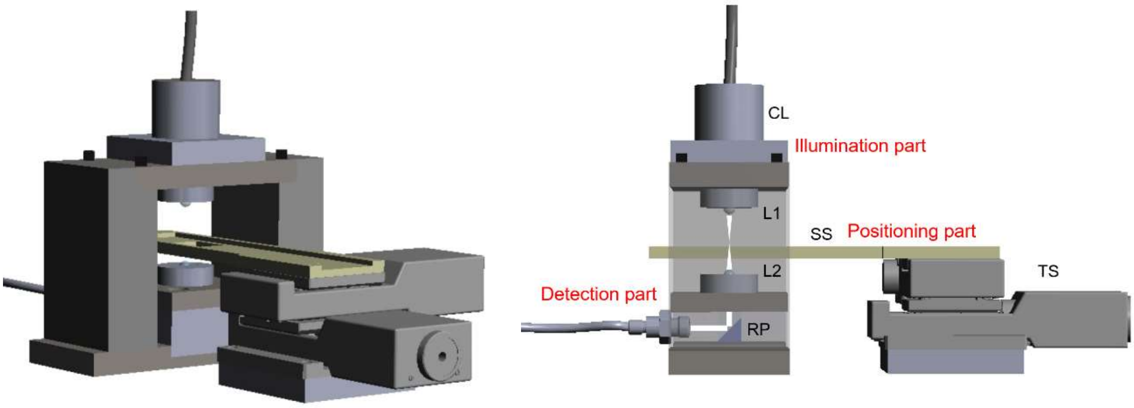

Fig. S1. (A) Two dimensional absorption spectrum measurement system and (B) its perspective side view: CL, collimating lens; L1, focusing lens; L2, collecting lens; RP, reflection prism; SS, sample stage; TS, two dimensional translation stage.

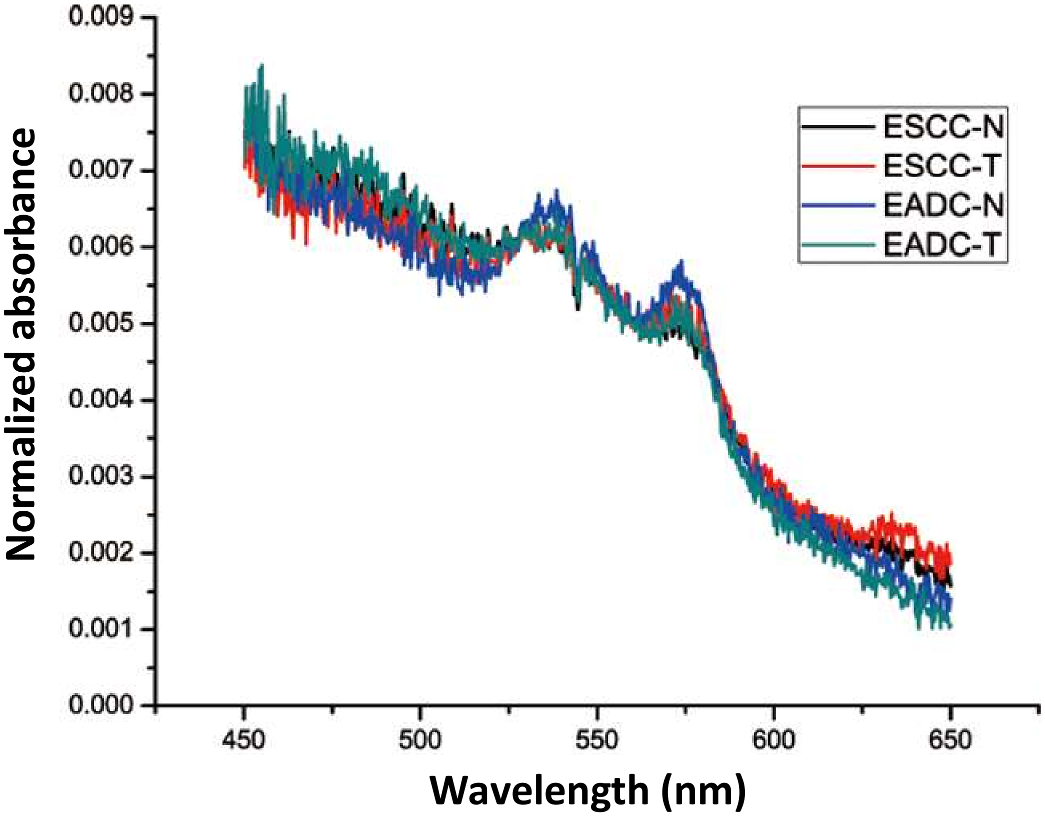

Fig. S2. Median absorption spectra of normal and tumor tissues of both ESCC and EADC patients. ESCC-N, ESCC-normal; ESCC-T, ESCC-tumor; EADC-N, EADC-normal; EADC-T, EADC-tumor.

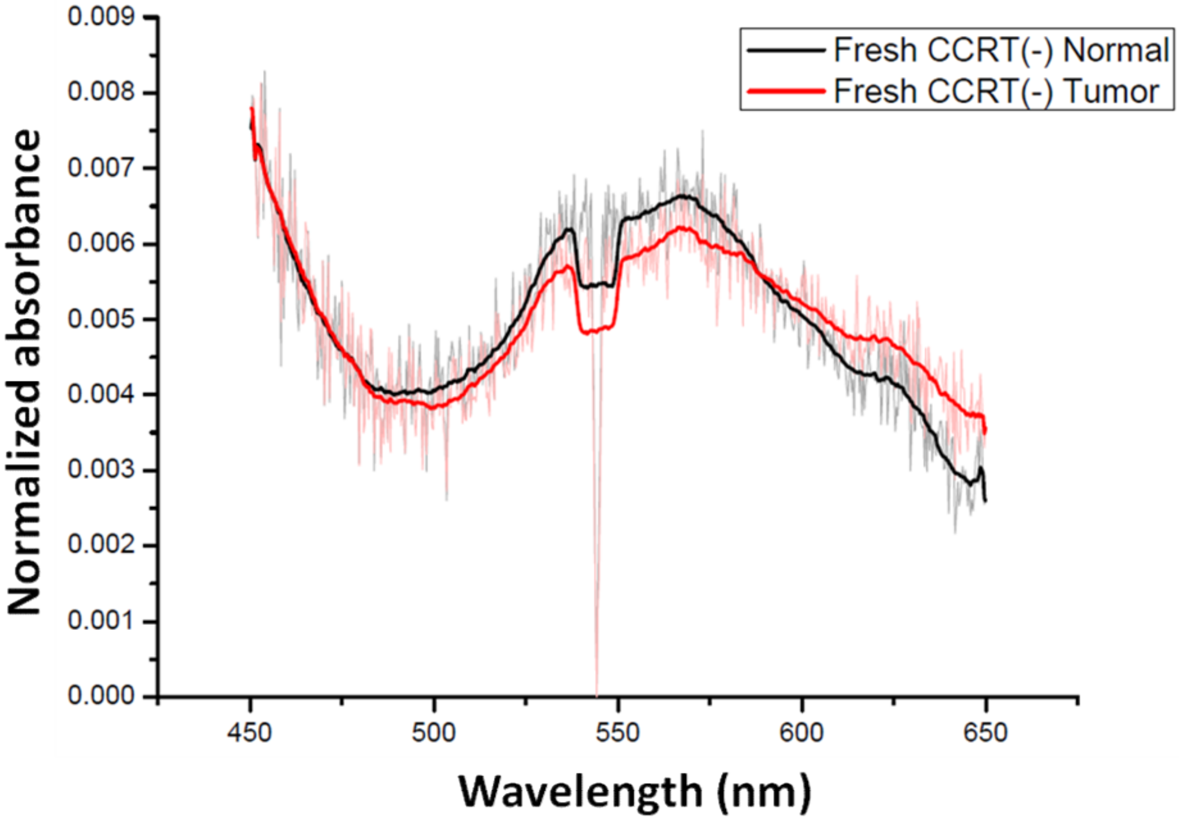

Fig. S3. Median absorption spectra of fresh normal and tumor tissues from CCRT (-) patients analyzed using the reflection mode of TDAS.

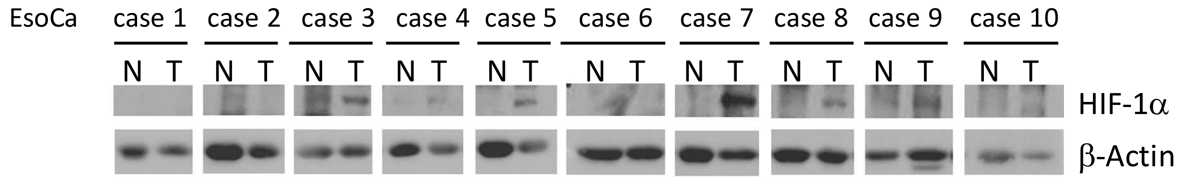

Fig. S4. HIF-1α expression in adjacent normal and cancerous esophageal tissue from 10 patients with esophageal cancer (EsoCa). Tissue protein was extracted by super lysis buffer (containing 3% SDS, 2 M urea, and 2% 2-mercaptoethanol).The expression of HIF-1α was analyzed by SDS-PAGE and western blotting with specific antibody (Cell Signaling Technology, 3716S). β-actin served as a loading control.

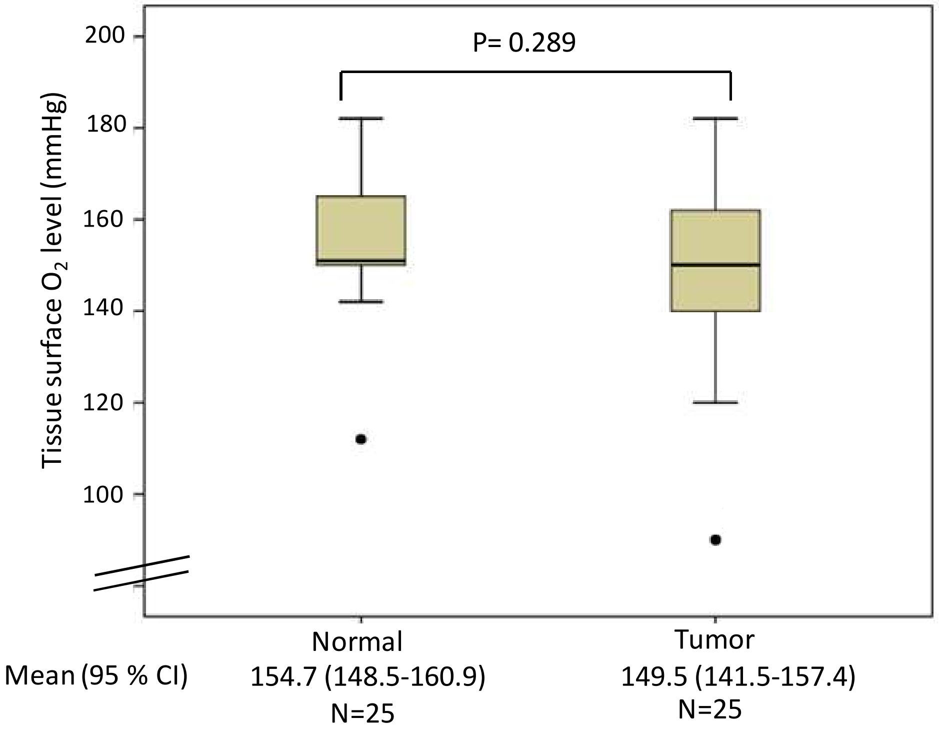

Fig. S5. Surface oxygen level of normal and cancerous tissue (N=25) measured by the OxyLite^{TM} Pro (Oxford Optronix) using an Oxygen-only bare-fibre sensor.
